# Antisense Oligonucleotides Capable of Promoting Specific Target mRNA Reduction via Competing RNase H1-Dependent and Independent Mechanisms

**DOI:** 10.1371/journal.pone.0108625

**Published:** 2014-10-09

**Authors:** Timothy A. Vickers, Stanley T. Crooke

**Affiliations:** Department of Core Antisense Research, ISIS Pharmaceuticals, Inc., Carlsbad, California, United States of America; International Centre for Genetic Engineering and Biotechnology, Italy

## Abstract

Antisense oligonucleotides (ASOs) are most commonly designed to reduce targeted RNA via RNase H1-dependent degradation. In this paper we demonstrate that cellular proteins can compete for sites targeted by RNase H1-dependent ASOs. We further show that some ASOs designed to mediate RNase H1 cleavage can, in certain instances, promote target reduction both by RNase H1-mediated cleavage and by steric inhibition of binding of splicing factors at a site required for efficient processing of the pre-mRNA. In the latter case, RNase H cleavage was prevented by binding of a second protein, HSPA8, to the ASO/pre-mRNA heteroduplex. In addition, using a precisely controlled minigene system, we directly demonstrated that activity of ASOs targeting sites in introns is strongly influenced by splicing efficiency.

## Introduction

Several mechanisms are known by which short synthetic oligonucleotides can be used to modulate gene expression in mammalian cells [1]. These antisense mechanisms require binding of the oligonucleotide to the targeted RNA and are broadly classified as cleavage-dependent or occupancy-only mechanisms. Perhaps the most commonly exploited antisense mechanism is RNase H-dependent degradation of the targeted RNA [1], [2]. Human cells express two types of RNase H: RNase H1 and RNase H2. Human RNase H1 is active as a single peptide, whereas RNase H2 is a heterotrimeric enzyme [3], [4]. Both enzymes are thought to play a role in DNA replication and repair, but additional biological functions are likely for both. Both RNase H isozymes recognize an RNA-DNA heteroduplex and cleave the RNA strand, resulting in a 5′-phosphate on the product and release of the intact DNA strand. RNase H1 is the enzyme responsible for mediating the target RNA cleavage directed by antisense oligonucleotides (ASOs) containing five or more consecutive DNA nucleotides [5]. Human RNase H1 binds to the RNA-DNA heteroduplex through an RNA binding domain located on the N terminus of the protein and cleaves the RNA 7 to 10 nucleotides, approximately one helical turn, from the 5′-end of the duplex region. The RNase H1 mechanism has been broadly exploited as both a research tool and a human therapeutic [6].

RNase H-independent, occupancy-only mechanisms have also been reported for antisense oligonucleotide drugs that have been chemically modified to result in loss of the ability to activate RNase H cleavage. Most mRNAs undergo a complex series of processing steps including splicing, polyadenylation, and addition of the 7 mG5′-cap structure [7]. Antisense mechanisms that rely on binding to the target RNA but not its degradation generally interfere in pre-RNA metabolism at one of these processing steps. For example, ASOs that bind to sequences required for splicing may prevent binding of necessary splicing factors or may physically prevent the cleavage reactions required for splicing, resulting in inhibition of the production of the mature mRNA [8], [9]. A wide range of non-DNA-like chemical modifications have been incorporated into ASOs, and the type of modification influences the mechanism of action. For example, an ASO with 2′-methoxyethyl (MOE) nucleotides promotes nearly complete inclusion of SMN2 exon 7–an alternatively spliced exon–by binding to an intronic splicing silencer in intron 7 [10]. In contrast, an ASO of the same sequence with 2′-fluoro- ribose nucleotides has the opposite effect; it induces skipping of exon 7 [11]. Translation can be inhibited using ASOs designed to bind to the translation initiation codon [12]; however, optimal inhibition is effected by binding at the 5′-cap in RNAs that have significant 5′-untranslated regions [13], [14]. ASOs targeted to poly A signals and/or sites in the 3′-terminal region of pre-mRNA have been shown to inhibit polyadenylation and destabilize the RNA [15]. In addition to 5′-capping and 3′-adenylation, there are clearly other sequences in the 5′- and 3′-untranslated regions of mRNA that impact stability, localization, and translatability [12]. ASOs have also been utilized to bind to a target RNA and disrupt RNA structures, interfering with the regulatory role provided by the structure [16].

Inhibition of splicing involves blocking or recruitment of proteins to splice-regulatory sites. Similarly, the RNase H1 mechanism requires recruitment of RNase H1 and associated proteins to the targeted cleavage site [17]. Other proteins have been also shown to interact with the ASO/RNA heteroduplexes to influence ASO activity [18]. In the current study we evaluated whether cellular proteins compete for sites targeted by RNase H1-dependent ASOs. We identify DNA-like ASOs that are capable of mediating RNase H1 cleavage and of displacing factors required for efficient splicing of the pre-mRNA. Our data suggest that there is a competition for the pre-mRNA-ASO heteroduplex between RNase H1 and heat shock 70 kDa protein 8 (HSPA8). When HSPA8 is bound to the pre-mRNA-ASO heteroduplex, the site becomes inaccessible to RNase H1; however reduction of spliced mRNA can still be affected by displacing certain splicing factors from the pre-mRNA resulting in an inhibition of splicing. In addition, using a precisely controlled minigene system, we demonstrate directly that activity of ASOs targeting sites in introns is strongly influenced by splicing efficiency.

## Materials and Methods

### Preparation of antisense oligonucleotides

**Figure 1:**
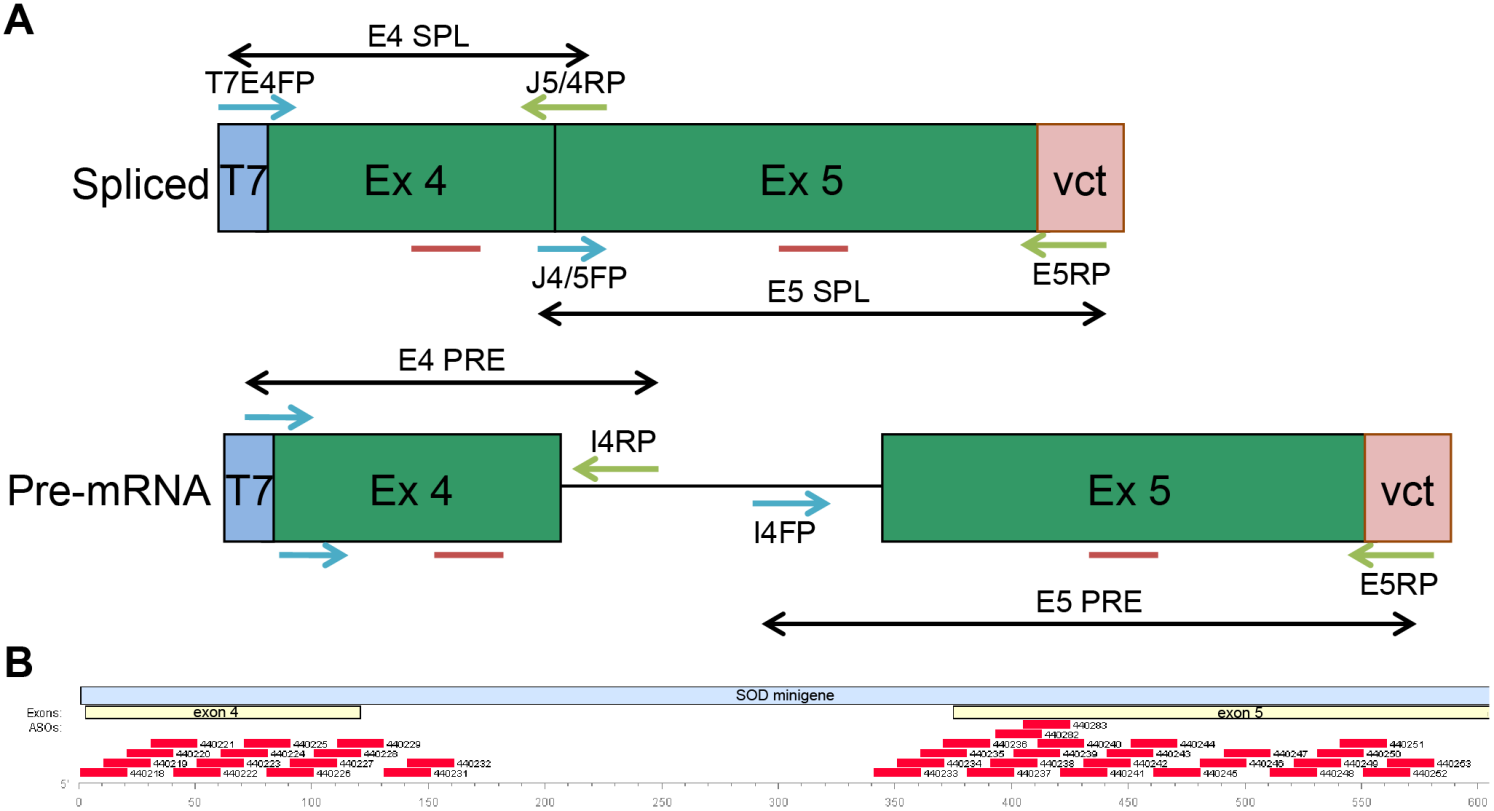
Tetracycline-inducible SOD1 minigene system. A) Strategy for quantification of SOD1 minigene expression by qRT/PCR. Minigene expression was distinguished from that of the endogenous SOD1 gene using forward or reverser primers homologous to pcDNA4 vector sequence. Spliced mRNA was distinguished from pre-mRNA using primers spanning the exon4/exon5 junction for amplification of the spliced mRNA and intron-specific primers for the pre-mRNA. The same TaqMan probe detected spliced and pre-mRNA. B) Relative position of 20-mer MOE gapmer ASOs on the SOD minigene transcript.

Synthesis and purification of phosphorothioate/2′-MOE oligonucleotides was performed using an Applied Biosystems 380B automated DNA synthesizer as described previously [19]. All ASOs used were 20 bases in length. Each residue of each ASO was a phosphorothioate, and there were 2′-*O*-methoxyethyl substitutions at the positions 1–5 and 16–20 for gapmers or at every position in 2′MOE ASOs. The sequences are listed in Table 1 and relative locations on the transcript are depicted in Figure 1B.

**Table 1:** Sequences of ASOs used in the study.

IsisNo	Sequence
440218	CCCAAGTCTCCAACATGCCT
440219	AGTCACATTGCCCAAGTCTC
440220	CTTTGTCAGCAGTCACATTG
440221	GCCACACCATCTTTGTCAGC
440222	AGACACATCGGCCACACCAT
440223	AATCTTCAATAGACACATCG
440224	GAGATCACAGAATCTTCAAT
440225	TCCTGAGAGTGAGATCACAG
440226	TGCAATGGTCTCCTGAGAGT
440227	CGGCCAATGATGCAATGGTC
440228	CACCAGTGTGCGGCCAATGA
440229	GAAAACTTACCACCAGTGTG
440230	ATCCTTTTATGAAAACTTAC
440231	TTTTATGCATATCCTTTTAT
440232	TTAGAAGAAGTTTTATGCAT
440233	AAGAAGATAACTTTGGGCTG
440234	AAAAAATTTTAAGAAGATAA
440235	ATGGACCTGTAAAAAATTTT
440236	CTGCTTTTTCATGGACCTGT
440237	CCCAAGTCATCTGCTTTTTC
440238	TCCACCTTTGCCCAAGTCAT
440239	TTTCTTCATTTCCACCTTTG
440240	GTCTTTGTACTTTCTTCATT
440241	AGCGTTTCCTGTCTTTGTAC
440242	AACGACTTCCAGCGTTTCCT
440243	CCACAAGCCAAACGACTTCC
440244	CCCAATTACACCACAAGCCA
440245	ATTGGGCGATCCCAATTACA
440246	CTACATCCAAGGGAATGTTT
440247	GGGCCTCAGACTACATCCAA
440248	AGGATAACAGATGAGTTAAG
440249	TACAGCTAGCAGGATAACAG
440250	GATACATTTCTACAGCTAGC
440251	TGTTTATCAGGATACATTTC
440252	CAGTGTTTAATGTTTATCAG
440253	TTTAAGATTACAGTGTTTAA
440282	TTTCCACCTTTGCCCAAGTC
440283	GTACTTTCTTCATTTCCACC
440238*	TagAtCTTTGCttAAGTCAT
440282*	TTTagAtCTTTGCttAAGTC
440283*	GTACTTTCTTCATTTagAtC

All RNase H-dependent ASOs used for target mRNA reduction were 20 bases in length, all linkages were phosphorothioate and 2′-*O*-methoxyethyl residues were incorporated at the positions 1–5 and 16–20. An analog of each ASO was also synthesized with 2′-*O*-methoxyethyl at each position.

*Asterisks indicate ASOs targeting SOD/TO-AB mutant sites and base changes relative to parent ASO are indicated by lower case letters.

### TET-inducible mini gene system

Construction of the SOD1 minigene, pcDNA_SOD1, and generation of stable minigene cell lines was described previously [20]. A splice-defective mutant, pcSOD187M/TO, in which the native U1 consensus sequence, UG **GU**AAGU was converted to UG GUUGGG to prevent binding of U1 snRNP at the 5′ splice site, was generated by site directed mutagenesis using a QuikChange Lightning SDM Kit (Agilent Technologies) with primers W187F 5′-CATCATTGGCCGCACACTGGTGGTTGGGTTTCATAAAAGGATATGCATAAAAC-3′ and W187R 5′-GTTTTATGCATATCCTTTTATGAAACCCAACCACCAGTGTGCGGCCAATGATG-3 according to the manufacturer’s protocol.

T-REx-293 and T-REx-HeLa cells were purchased from Invitrogen and cultured in DMEM supplemented with 10% fetal calf serum, 0.1 µg/ml streptomycin, 100 units/ml penicillin, and 5 µg/ml blasticidin. Plasmids pcSOD1/TO and pcSOD187M/TO were transfected into T-REx-293 cells using Effectene transfection reagent according to the manufacturer’s protocol (Qiagen). Cells in which the minigene was stably integrated were selected in DMEM media containing 250 µg/ml zeocin. Zeocin-resistant colonies were expanded then tested for induction of expression by tetracycline (TET) using qRT/PCR. Cell lines overexpressing *E. coli* RNase H were generated as described previously [20].

### ASO/siRNA treatment and evaluation of target reductionand qRT/PCR

SOD/TO and SOD187M/TO cells were seeded in 96 well plates at ∼50% confluency then treated the following day with the indicated concentrations of ASO in Opti-MEM media (Invitrogen) containing 5 µg/ml Lipofectamine 2000 (Invitrogen) for 4 h, as described previously [8]. Following transfection, cells were washed once with PBS then fed with fresh growth media containing 1 µg/ml TET to induced minigene transcription. Total RNA was purified using an RNeasy 3000 BioRobot (Qiagen), and mRNA levels were assessed by qRT/PCR performed essentially as described elsewhere [21]. Briefly, 10 µl of total RNA was analyzed in a final volume of 50 µl containing 200 nM gene-specific PCR primers, 0.2 mM of each dNTP, 75 nM fluorescently labeled oligonucleotide probe, 5 µl RT-PCR buffer, 5 mM MgCl_2_, 2 U of Platinum Taq DNA Polymerase (Life Technologies), and 8 U of RNase inhibitor. Reverse transcription was performed for 30 min at 48°C followed by PCR: 40 thermal cycles of 30 s at 94°C and 1 min at 60°C using an ABI Prism 7700 Sequence Detector (Applied Biosystems). To avoid artifacts based upon well-to-well variation in cell number, mRNA levels were normalized to the total amount of RNA present in each reaction as determined by the Invitrogen Ribogreen assay [22]. Primers were designed to specifically amplify spliced or pre-mRNA, and both were detected by the same probe. To avoid amplification of endogenous SOD1, primers included vector sequence unique to the mini gene [20]. Sequences of primers and probes for qRT/PCR can be found in Table S1.

For RNase H reduction experiments, 10^7^ cells were seeded in 10-cm plates and treated for 4 h with 25 nM siRNA (Ambion Silencer siRNA, # s48358) in OptiMem media with 6 µg/ml Lipofectamine RNAiMax. Cells were allowed to recover for 4 h in complete media then seeded in 6-well dishes at 350,000 cells/well. The following day cells were treated with ASOs as detailed above. Total RNA was purified using an RNeasy mini prep kit (Qiagen) according to the manufacturer’s protocol. RNase H1 and SOD/TO mRNA reduction were assessed by qRT/PCR. For Northern blots, 5 ug/lane RNA was separated on a 1.2% agarose gel containing 1.1% formaldehyde, then transferred to Hybond membranes (Amersham Biosciences). SOD/TO or B-actin cDNAs were ^32^P random prime labeled using a Prime-a-Gene Labeling System according to the manufacturer’s protocol (Promega). Blots were hybridized at 39°C for 2 h in Rapid-hyb solution (Amersham Biosciences) then washed with 2X SSC containing 0.1% SDS at 39°C for 15 minutes, followed by 0.1X SSC containing 0.1% SDS at 60°C.

For hnRNP H/F reduction experiments, 4×10^6^ HeLa SOD/TO cells were seeded in 6 cm plates and treated for 4 hours with hnRNP F siRNA (Santa Cruz Biotechnology, sc-38272) or hnRNP H siRNA (sc-35579) at 30 nM in OptiMem media with 6 ug/ml Lipofectamine RNAiMax. Cells were allowed to recover overnight in complete media then the transfection was repeated. 96 well plates were then seeded with control or hnRNP-reduced cells at 9000 cells/well. The following day SOD minigene expression was induced by addition of 1 ug/ml TET. Cells were harvested at indicated times post TET addition and mRNA purified as detailed above. Expression levels of spliced SOD1 minigene RNA were determined by qRT/PCR with primer/probe set E5 SPL (Figure 1) and standards of known quantity.

Western blots were performed as described above with rabbit anti-hnRNP H (ABCAM ab10374) or rabbit anti-hnRNP F (ABCAM ab50982). For HSPA8 reduction 293 SOD/TO cells were treated with HSPA8 siRNA (sc-44263) as above. Western blots were performed with anti-HSPA8 (ABCAM ab79857) or anti-tubulin as a control. All control cells were treated with an siRNA targeting luciferase (Dharmacon P-002099-01-50).

### RNA pull-downs

Pull-down of RNA binding proteins was performed essentially as described previously [11] using a 39-nt 5′ biotin-labeled RNA corresponding to SOD_PD_C (5′-AGCAGAUGACUUGGGCAAAGGUGGAAAUGAAGAAAGUC-3′) or SOD_PD_M (5′-GGUACGUUUUGUCUCUGGUCCUUACUUAUACCGGUGUAC-3′). When present, 3 nmol of ASO was incubated with 1 nmol of biotinylated RNA in 1x siRNA buffer (Thermo Scientific) for 1 min at 90°C. Single-stranded or oligonucleotide-bound biotinylated RNA oligonucleotide was then mixed with 100 µl of prewashed Dynabeads MyOne Streptavidin C1 (Invitrogen) 1× siRNA buffer. RNA was bound to beads by incubation for 20 min at room temperature. Beads were then washed three times in BW buffer (10 mM Tris-HCl, pH 7.5, 1 mM EDTA, 2 M NaCl, and 0.01% Tween 20), then incubated with 25 µl HeLa nuclear extract (Promega), 1.6 mM MgCl_2_, 20 mM creatine phosphate, 500 µM ATP, 10 mM HEPES-KOH (pH 8) and 12.5 µl Buffer D (20 mM HEPES-KOH, pH 8, 20% (v/v) glycerol, 0.1 M KCl, 0.2 mM EDTA, 1 mM DTT, 0.5 mM phenylmethanesulfonyl fluoride). Mixtures were incubated with constant rotation for 45 min at 25°C. Beads were isolated by magnetic selection and washed three times with Buffer D containing 300 mM KCl. The beads were then incubated with a 50-µl mixture containing 1,000 U RNase I (Ambion) and 10 mM Tris-HCl (pH 8) for 40 min at 30°C with constant rotation. Eluted proteins were resolved on Novex 4–12% bis-Tris gels in 1X MOPS buffer or 3–8% Tris-acetate gels (Invitrogen). Gels were then silver stained using a ProteoSilver kit according to the manufacturer’s instructions (Sigma-Aldrich). Twenty percent of the released fraction was used for western blotting as described elsewhere [23] using mouse anti-γ-tubulin from Sigma-Aldrich (T5326), rabbit anti-hnRNP H (ABCAM ab10374), or mouse anti-hnRNPK (Origene TA307474).

## Results

### Effects of RNA processing on antisense oligonucleotide activity

To evaluate the effects of RNA processing on ASO activity, a minigene system precisely controlled by addition of tetracycline (TET) was utilized [20]. T-REx 293 cells stably expressing the construct were evaluated for TET-inducible expression of the minigene by qRT/PCR using the primers and probes outlined in Figure 1 and Table S1. Up-regulation in levels of pre-mRNA was detected almost immediately following addition of TET with maximal expression of approximately 300% uninduced control 45–60 minutes after addition of TET to the media (Figure S1A). Detectable levels of spliced mRNA lagged synthesis of the pre-mRNA by approximately15 minutes; however, induction was at least 20 fold and did not plateau until almost 4 h after addition of TET (Figure S1B).

A second splicing-defective minigene construct was also developed. Site directed mutagenesis was used to convert the canonical GUAAGU 5′ donor splice site of pcSOD/TO to GUUGGG. In T-REx 293 cells stably expressing the resulting plasmid, pcSOD/TO-187, the minigene RNA was transcribed, but splicing efficiency was greatly reduced and the majority of the pre-mRNA was retained in the nucleus (Figures S1C–F).

**Figure 2:**
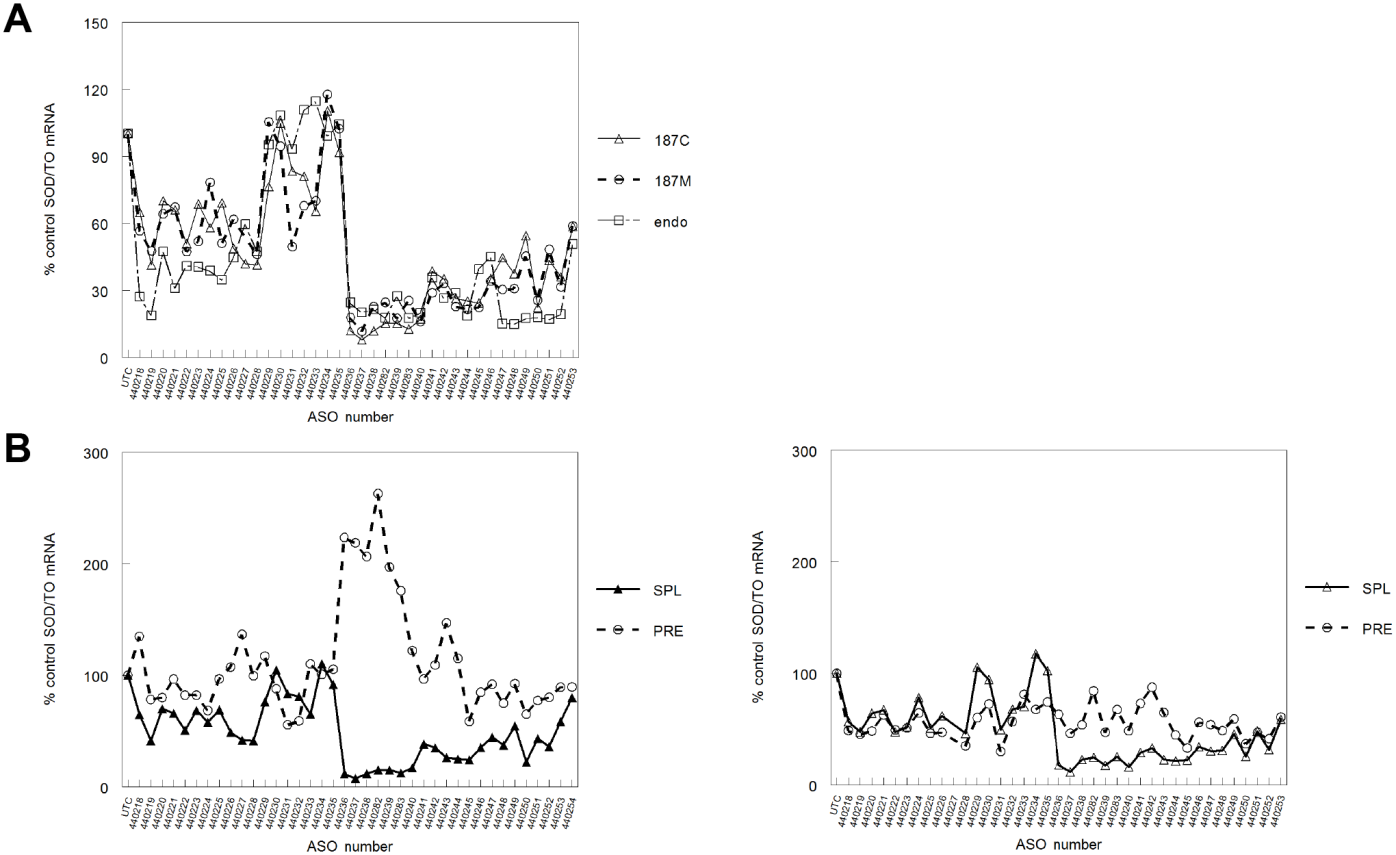
Activities of ASOs spanning the SOD1 minigene. A) SOD/TO or SOD/TO-187 cells were transfected with each of a series of 38 ASOs. Following ASO treatment, reduction of *SOD1* transcript expressed from the minigene and of endogenous *SOD1* was evaluated by qRT/PCR using primers and probes specific for the minigene encoded transcript or endogenous *SOD1*. Data are presented as percent spliced mRNA relative to that in mock-treated control cells. Δ = SOD/TO, ○ = SOD/TO-187, □ = endogenous SOD1. B) SOD/TO cells were transfected with ASO at 60 nM. Following transfection and tetracycline induction, expression of spliced and pre-mRNA was evaluated by qRT/PCR using minigene transcript-specific primers and probes. Data are presented as percent spliced mRNA or pre-mRNA relative to the same transcript in mock-treated control cells. Δ = pliced, ○ = pre-mRNA. C) As in panel B, but with SOD/TO 187 cells.

A series of 38 antisense oligonucleotides (ASOs) targeted to the SOD1 minigene was screened for activity in both SOD/TO and SOD/TO-187 cell lines. All ASOs were chimeric “gapmers”, 20 bases in length with phosphorothioate backbones and 2′-*O*-methoxyethyl (2′MOE) substitutions at positions 1–5 and 16–20. ASOs were transfected into cells in the presence of cationic lipid for 4 h at a concentration of 50 nM. Following the transfection, minigene expression was induced by incubating cells for 3 h in complete media containing 1 µg/ml TET. mRNA levels were assessed by qRT/PCR using the primer probe sets shown in Figure 1. To avoid possible false positives due to the presence of the ASO in the amplicon, the exon 5 primer/probe set (E5 SPL) was used for ASOs directed to exon 4 sequences and the exon 4 primer/probe set (E4 SPL) was used for ASOs complementary to exon 5. Effects of the ASO on the endogenous *SOD1* message was evaluated using a primer/probe set specific for exon 2 of *SOD1*. Patterns of mRNA reduction were remarkably similar for the control and donor-site mutated genes despite the differences in processing and cellular localization of the RNAs (Figure 2A). Patterns of mRNA reduction for the endogenous *SOD1* were also similar to that of the mini-gene, with slightly more activity observed in exon 4 and in exon 5 with ASOs 440247-52, but with less activity in ASOs targeting the intron (ASOs 440228-36).

The effects of ASO administration on minigene pre-mRNA levels were also evaluated. In the SOD/TO cell line, a significant increase in pre-mRNA levels was observed upon treatment with ASOs targeting the region of the 5′ splice site (Figure 2B, ASOs 440237-9, 440282 and 440283). A similar increase in pre-mRNA levels was not observed in the SOD/TO-187 cell line where the level of reduction of spliced and pre-mRNA was more closely correlated (Figure 2C), suggesting that in the parental cell line at least a portion of the observed reduction in mRNA levels may be due to effects of the ASO on splicing efficiency. In the SOD/TO-187 cell line splicing efficiency is reduced relative to that in the parental cell line and therefore most activity results from RNase H-mediate cleavage.

**Figure 3:**
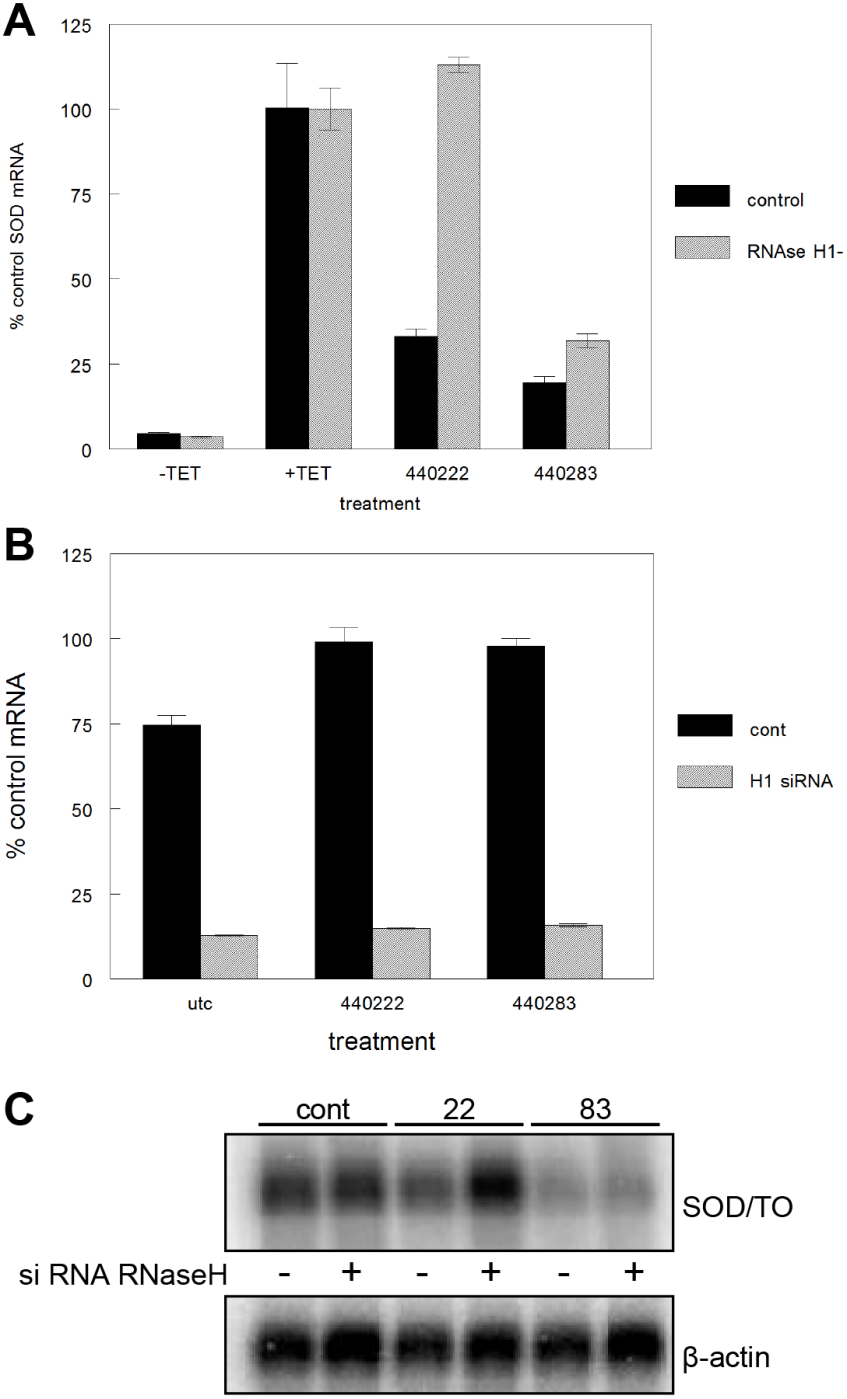
Effect of RNase H1 reduction on activity of SOD1 minigene ASOs. A) SOD/TO cells were treated with an siRNA targeting human RNase H1. After 48 hours, RNase H-deficient and control cells were transfected with SOD1 ASOs at 50 nM. Following TET induction, expression of minigene-derived spliced mRNA was assessed by qRT/PCR. Expression is shown relative to mock-treated control. Solid bars, SOD/TO cells; striped bars, SOD/TO treated with siRNA targeting gene encoding RNase H1. B) Northern analysis of levels of SOD/TO minigene transcript. Cells were treated as in panel A.

To explore the possibility that the observed reductions in spliced mRNA result from different mechanisms, several experiments were performed to evaluate the RNase H1 dependence of activity in both control and mutant cell lines. An siRNA targeting human RNase H1 was used to reduce RNase H1 protein levels in the SOD/TO cell line. Following reduction of RNase H1, cells were treated with an ASO that mediates RNase H1 cleavage only (440222) or with an ASO capable of blocking splicing and increasing pre-mRNA (440283). The next day, mRNA levels were assessed by qRT/PCR. For ASO 440222, approximately 65% reduction of the minigene mRNA was observed in cells not treated with siRNA (Figure 3A, solid bars). However, in cells in which RNase H1 levels were reduced (Figure 3B), activity of the same ASO was completely ablated (Figure 3A, striped bars). Treatment with ASO 440283 resulted in approximately 80% reduction in target RNA in untreated cells and RNase H1 reduction had very little effect on the activity of this ASO. Northern blots were performed to confirm these observations. In control cells, treatment with ASO 440222 resulted in significant reduction in the SOD/TO mRNA, whereas in RNase H1-depleted cells the same ASO was inactive (Figure 3B, compare lanes 3 and 4). In contrast, treatment with ASO 440283 resulted in significant reduction of the minigene mRNA in both control and RNase H1-depleted cells (Figure 3B, lanes 5 and 6).

**Figure 4:**
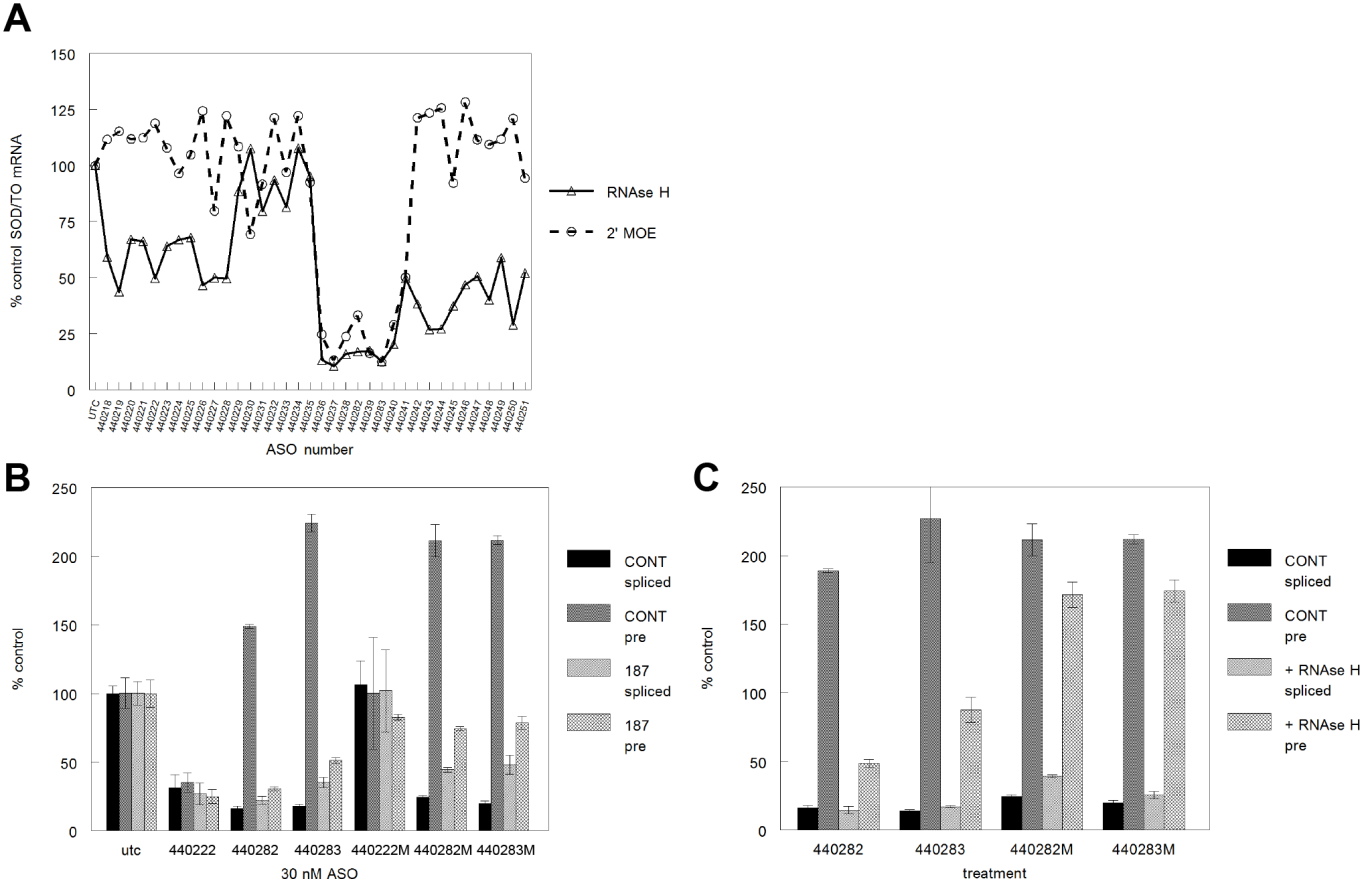
Activities of RNase H-independent 2′MOE ASOs. A) SOD/TO cells were transfected with a series of 38 2′MOE ASOs and gapmer ASOs of the same sequences (these gapmers were also tested in a separate experiment shown in Figure 2). Following ASO treatment, levels of SOD1 minigene mRNA were analyzed by qRT/PCR. Data are presented as percent spliced mRNA in treated vs. mock-treated cells. Δ = gapmer ASOs, ○ = 2′MOE ASOs. B) SOD/TO or SOD/TO-187 cells were transfected with 50 nM gapmer ASOs 440222 or 440282 and 2′MOE ASOs of the same sequence. Levels of spliced and pre-mRNA were assessed by qRT/PCR using the primer/probes depicted in Figure 1. Activity is presented as percent expression relative to levels in mock-transfected cells. Solid bars, SOD/TO spliced mRNA; grey bars, SOD/TO pre-mRNA; striped bars, SOD/TO-187 spliced mRNA; hatched bars, SOD/TO-187 pre-mRNA. C) SOD/TO or SOD/TO-H1 cells were transfected with 30 nM gapmer ASOs 440282 or 440283 and 2′MOE ASOs of the same sequence. Levels of SOD1 minigene spliced and pre-mRNA were assessed by qRT/PCR as described above. Solid bars, SOD/TO spliced mRNA; grey bars, SOD/TO pre-mRNA; striped bars, SOD/TO-H1 spliced mRNA; hatched bars, SOD/TO-H1 pre-mRNA.

Many oligonucleotide modifications have been designed that do not support RNase H1 activity [24]–[26]. For example, ASOs fully modified with 2′MOE do not induce RNase H1-mediated degradation of target RNAs, yet maintain high affinity and target specificity [15], [27]. An additional set of 38 fully modified 2′MOE ASOs were synthesized and screened for activity in the SOD/TO cell line. In contrast to RNase H1-dependent ASOs, the 2′MOE ASOs were inactive except in the region just downstream of the 5′ splice site (Figure 4A).

Activities of certain ASOs were further evaluated in both SOD/TO and SOD/TO-187 cell lines. ASO 440222 was active in both cell lines, effectively reducing mature and pre-mRNA, whereas treatment with the 2′MOE analog, 440222M, did not result in reduction of either spliced or pre-mRNA in either cell line (Figure 4B). In contrast, both ASOs 440282/3 and the 2′MOE analogs, ASOs 440282/3M effectively reduced levels of spliced RNA in the SOD/TO cell line with a corresponding increase in pre-mRNA. In contrast to ASO 440222, 440282 was less active in SOD/TO-187 than SOD/TO cells and ASO 440282M was even less active in the SOD/TO-187 cells. In addition, upon treatment with ASOs 440282/3 and ASOs 440282/3M, rather than an increase in pre-mRNA levels, pre-mRNA levels in SOD/TO-187 cells were either reduced or unchanged. These data suggest that ASOs 440282/3 as either gapmers or 2′MOEs reduce splicing efficiency resulting in accumulation of pre-mRNA and ultimate reduction in spliced RNA. Since the SOD/TO-187 cells are splice-deficient compared to the parent cells, the effect is less pronounced.

These data also suggest that competition exists between RNase H1-dependent and non-RNase H1 mechanisms. This possibility was explored by overexpressing *E. coli* RNase H in both minigene cell lines. The *E. coli rha* gene was previously subcloned into the vector pcDNA 3.1 to allow stable expression in mammalian cells [20]. SOD ASOs were screened for activity in cells that stably overexpress RNase H. Although overexpression of RNase H significantly diminished the levels of pre-mRNA accumulation resulting from treatment with ASOs 440282 and 440283, pre-mRNA levels remained elevated in cell lines overexpressing RNase H when treated with 440282M and 440283M (Figure 4C). These data indicate that for RNase-H ASOs, an increase in RNase H levels can overcome effects of the competing non-RNase H mechanism.

### Antisense binding at the 440282/3 site leads to disruption/recruitment of splicing factors

**Figure 5:**
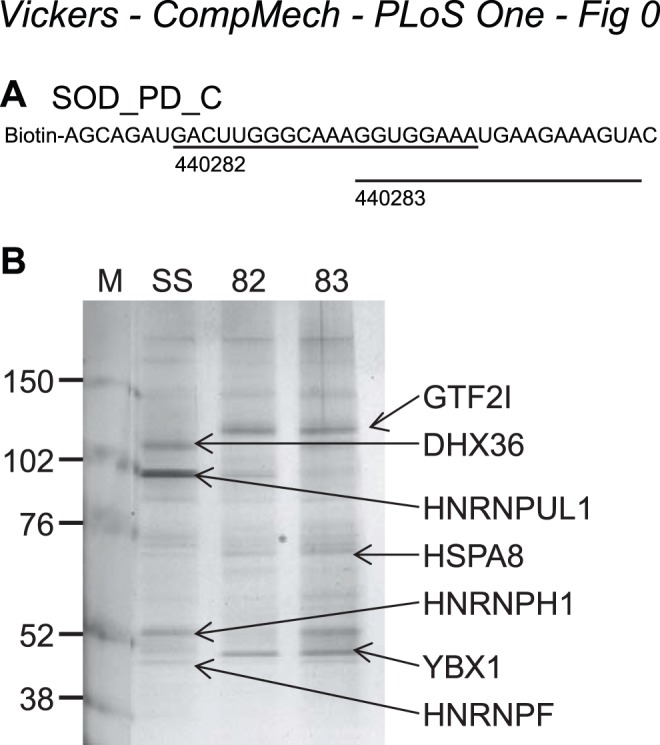
Identification of proteins that interact with *SOD1* mRNA in the region complementary to ASOs 440282 and 440283. A) Sequence of the 5′-biotinylated 39-mer SOD1 exon 5 RNA fragment. Binding sites for ASOs 440282 and 440283 are indicated. B) Silver-stained gel of proteins eluted from single-stranded RNA (SS) and from RNA hybridized to ASO 440282 or ASO 440283. RNA was incubated in the presence or absence of ASOs and then with HeLa cell nuclear extract. The beads were washed, and proteins released by RNase I digestion were identified by MS. Identities are presented as HGNC Gene symbols. M = marker, SS = RNA with no ASO, 82 = RNA/440282 duplex, 83 = RNA/440283 duplex.

The mechanism by which the ASOs reduce mRNA levels in the absence of RNase H1 cleavage was next explored. RNA pull-down experiments were conducted with an RNAse H gapmer ASO/RNA duplex. A 5′-biotinylated 39-mer with the sequence of a fragment of the SOD1 exon 5 (+12 to +50), which includes the 440282 and 440283 binding sites, was attached to streptavidin-coated magnetic beads (Figure 5A). Immobilized RNA was incubated in the presence or absence of ASO and then in HeLa cell nuclear extract. The beads were washed, and proteins released from the ASO/RNA complexes by RNase I digestion were resolved on a gel and visualized by silver staining. Protein bands were excised from the gel and identified by electrospray mass spectrometry analysis. In the absence of bound ASO, hnRNPs H, F, and UL1 and helicase DHX36 bound the RNA (Figure 5B, lane 2). Binding of DHX36 and hnRNPs H and F was diminished in the presence of gapmer 440282 (lane 3), but not gapmer 440283 (lane4), whereas binding of hnRNP UL1 was abrogated by both ASOs. Several proteins were also identified that were present only when either ASO was bound to the RNA; these included GTF2I, YBX1, HSPA8, and hnRNP K (Figure 5B, lanes 3 and 4; Figure S2A). To confirm specificity of binding, immunoblot experiments were performed (Figure S2B). Similar results were obtained when 2′MOE ASOs were used rather than gapmer ASOs.

**Figure 6:**
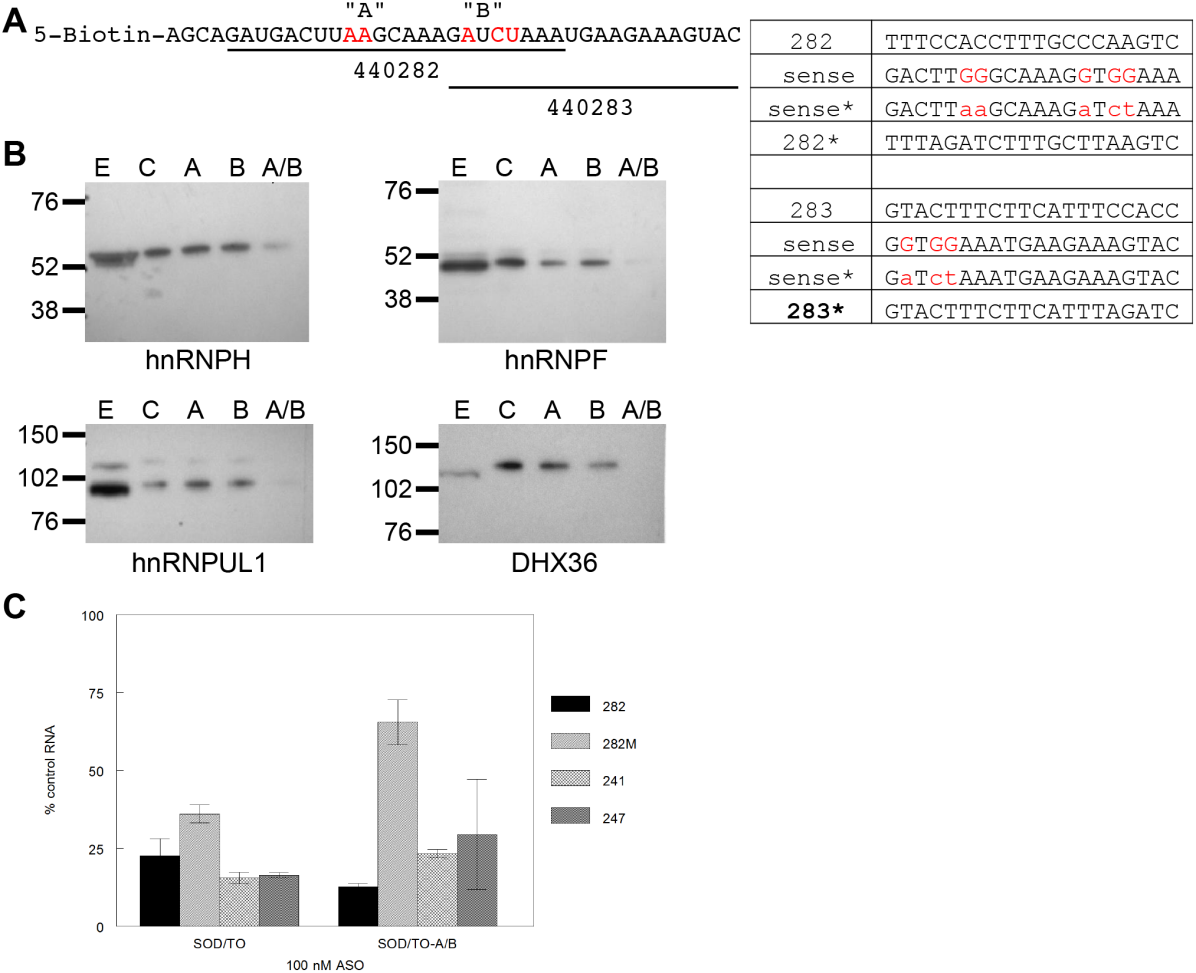
Effect of mutations at the 440282/440283 site on protein binding. A) 5′-biotinylated 39-mer SOD1 exon 5 RNA fragment was mutated at either or both of two putative hnRNP H/F binding sites. Nucleotide changes from control sequence (C) are indicated in red. ASOs 440282* and 440283* have complementary mutations. B) Proteins bound to control or mutated RNA/ASO hybrids were released by digestion with RNAse I. Eluted proteins were resolved on protein gels and western blots carried out using the indicated antibodies. C) A and B site mutations were introduced into the SOD/TO minigene by site-directed mutagenesis. Stable minigene cells lines were treated with ASO 440282* (solid bars) or with 2′MOE 440282* (striped bars) and with ASOs 440241 (hatched bars) and 440247 (grey bars) which lie outside of the mutated region. Data are presented as percent spliced minigene mRNA reduction relative to mock treated controls.

Unlike other hnRNP proteins which have a range of affinities for different ribonucleotide homopolymers and single-stranded DNA, hnRNP F and hnRNP H bind only to poly(rG) *in vitro* [28]. We therefore mutated two potential hnRNP H/F binding sites within the region containing the binding sites of ASOs 440282 and 440283. RNA pull-down experiments were conducted with the 5′-biotinylated 39-mer SOD1 exon 5 RNA fragment mutated at site A only, site B only, or both sites A and B (Figure 6A). Proteins released from the beads by RNase I digestion were separated by SDS PAGE and binding of specific proteins was evaluated by western blot (Figure 6B). In the cases of hnRNP H and hnRNP UL1, mutation at either site A or site B had no effect on protein binding relative to binding to the control capture RNA, whereas binding of hnRNP F and DHX36 was diminished, but not eliminated, by the mutation at either site. In contrast, binding of each of these proteins was virtually eliminated with the double mutation at the A and B sites. Each of these hnRNPs has been shown to be associated with pre-mRNAs in the nucleus of mammalian cells, and these proteins appear to influence pre-mRNA processing [29]. In addition, DHX36 has been implicated in a number of cellular processes involving alteration of RNA secondary structure, including spliceosome assembly [30]. Our data suggest that ASO 440282 reduces splicing efficiency by blocking access of these hnRNPs to the transcript, resulting in accumulation of pre-mRNA and a ultimate reduction in levels of spliced RNA.

To explore this possibility, the same mutations in each of these sites were introduced into the SOD/TO minigene by site-directed mutagenesis. A stable cell line harboring the mutated minigene construct, SOD/TO-AB, was treated with ASOs with base changes to result in complementarity to the mutated minigene (ASO 440282*). Both the gapmer and 2′MOE versions of ASO 440282 demonstrated significant activity when targeting the SOD/TO minigene (Figure 6C). Two other ASOs, 440241 and 440247, targeting different sites showed comparable activity. The gapmer ASO 440282* targeting the SOD/TO-AB mutant minigene reduced RNA transcript levels to a degree comparable to the gapmer 440282 targeting the parental minigene. However, the 2′MOE ASO 440282M* targeting the SOD/TO-AB mutant was significantly less active than its counterpart in the parental cells. The lack of activity with the 2′MOE ASO suggests that in the SOD/TO minigene construct, activity results, at least in part, from inhibition of hnRNP binding which leads to inefficient processing of the pre-mRNA. In agreement with this, levels of pre-mRNA were increased with the SOD/TO-AB as compared to the parent SOD/TO minigene (Figure S3).

**Figure 7:**
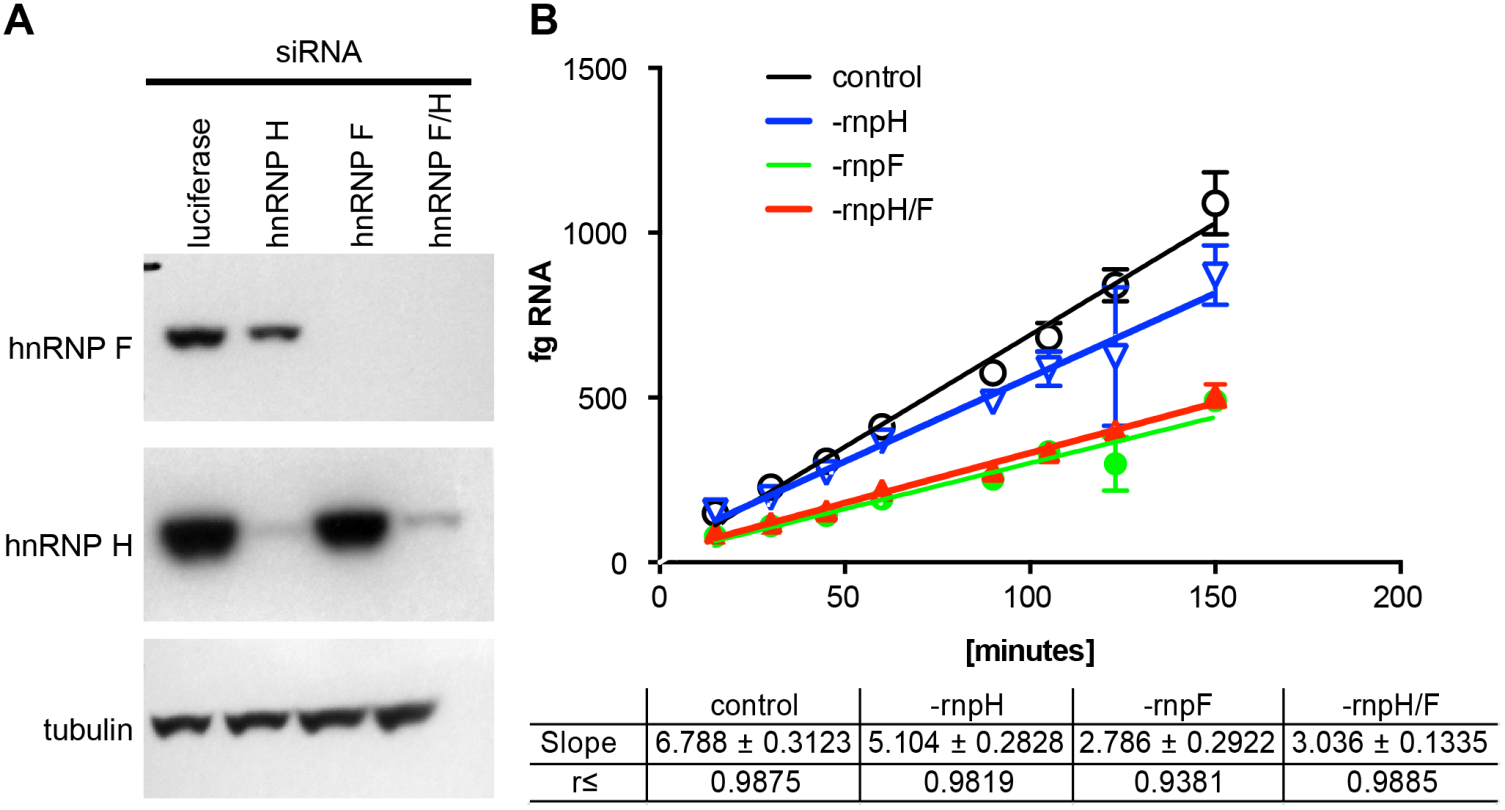
Reduction of hnRNPs decreases minigene splicing efficiency. HeLa SOD/TO cells were treated with siRNAs targeting luciferase, hnRNP H, or hnRNP F as detailed in Materials and Methods. After 48 hours hnRNP-reduced and control cells were seeded in 96 well plates at 5000 cells/well. A) Lysates were prepared from the remaining cells and Western blots carried out to assess reduction of hnRNP H and hnRNP F. B) Transcription was initiated by addition of TET to cells in 96-well plates. Total RNA was harvested at intervals between 10 minutes and 4 hours. qRT/PCR was performed using primer/probe set E4 SPL and standards of known quantity. Data are plotted as linear regression in fg mingene RNA/1000 cells vs. time (N = 4/time point).

To further confirm the involvement of hnRNPs H and F in mRNA processing, HeLa cells harboring the SOD/TO minigene construct were treated with siRNAs to reduce these proteins (Figure 7A). HeLa cells were used rather than 293s because siRNA mediated reduction of hnRNPs was much less efficient in 293 cells as compared to HeLa’s. However, similar RNAse H-independent ASO activity was observed in the HeLa SOD/TO cell line (Figure S4). Minigene transcription was induced by TET addition in hnRNP H/F-reduced as well as luciferase siRNA treated control cells, then terminated at regular intervals between 10 minutes and 4 hours. RNA expression levels of spliced SOD1 minigene RNA were determined by qRT/PCR with standards of known quantity. In control cells, the rate of formation of the spliced minigene transcript was 6.8±0.3 fg/minute (Figure 7B, black line). Reduction of hnRNP H lead to a slight decrease in the processing rate to 5.1±0.3 fg/minute. Reduction of hnRNP F or both hnRNPs F and H had a larger effect on RNA processing, lowering the rate by over half to 2.8±0.3 and 3.0±0.1 fg/minute respectively.

**Figure 8:**
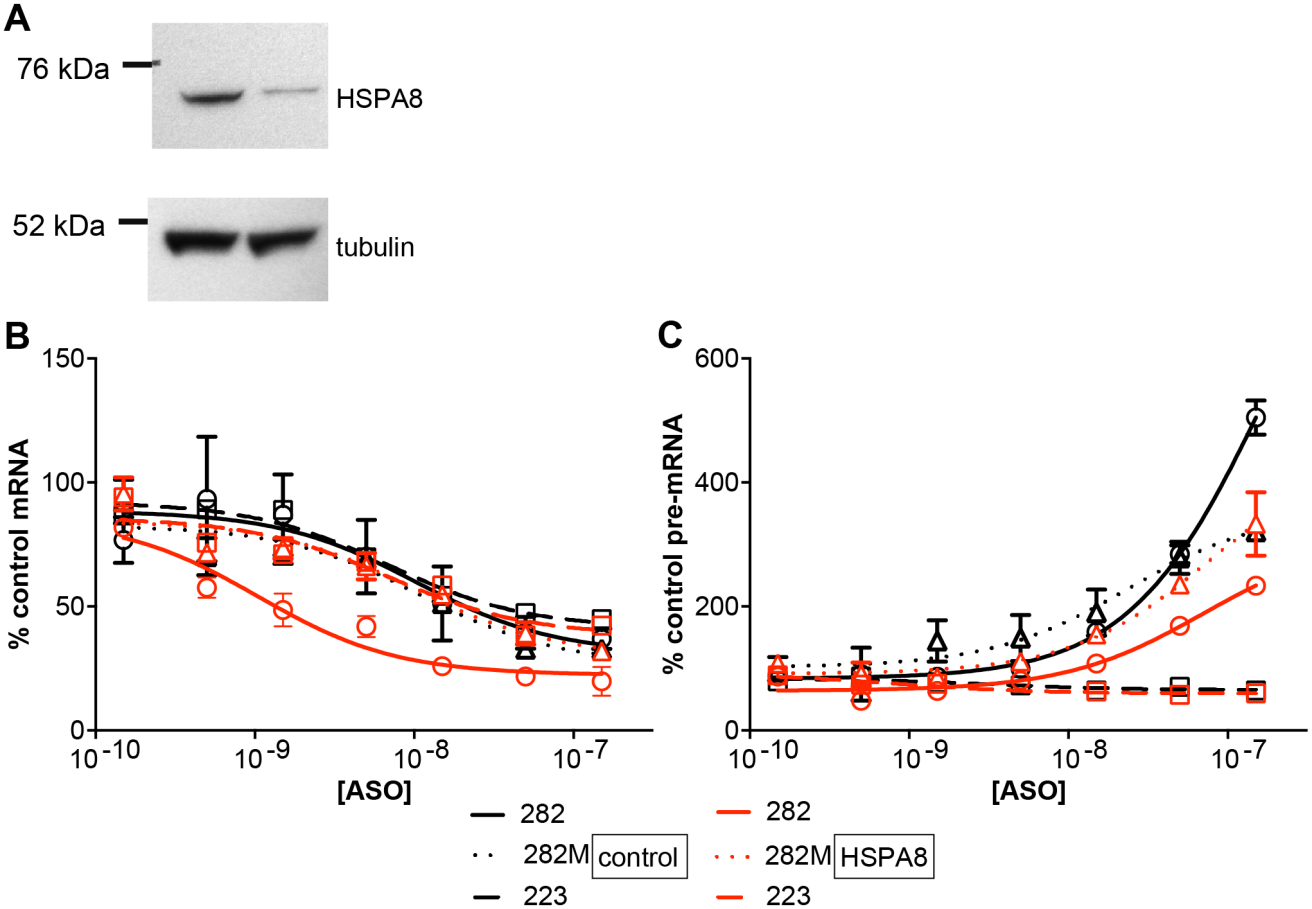
Effect of HSPA8 reduction on ASO 440282 activity. SOD/TO cells were treated with siRNA to reduce expression of HSPA8. After 48 hours, cells were transfected with ASOs at concentrations from 10 to 100 nM. Following a 4-hour transfection, minigene expression was induced by the addition of 1 µg/ml TET for 3 hours. A) Western blot of HSPA8 at 48 hours post-siRNA treatment. B) Levels of spliced mRNA relative to levels in mock-treated cells. Black lines = control cells, grey lines = HSPA8-deficient cells.

Finally, siRNAs were used to reduce the levels of HSPA8 in the SOD/TO cell line (Figure 8A). HSPA8-reduced and control cells without HSPA8 reduction were then treated with gapmer or 2′MOE versions of ASO 440282, and levels of spliced and pre-mRNA assessed by qRT/PCR. In the absence of siRNA, ASO 440282 decreased levels of spliced mRNA with an IC_50_ of approximately 23 nM (Figure 8B). ASO 440282M and control ASO 440223 had a similar IC_50_ values of 16 and 32 nM, respectively. Pre-treatment of cells with siRNA designed to reduce levels of HSPA8 had little effect on the activity of 2′MOE 440282M (17 nM) or control ASO 440223 (18 nM); however, the IC_50_ of the RNase H-activating ASO 440282 was reduced to 2 nM; an approximately 10 fold increase. Levels of pre-mRNA were also accessed following ASO treatment (Figure 8C). As observed previously, without siRNA pretreatment, the highest concentrations of ASOs 440282 and 440282M increased pre-mRNA levels by 3–5 fold, whereas control ASO 440223 treatment caused a slight decrease in pre-mRNA. Reduction of HSPA8 had no effect on pre-mRNA levels in cells treated with ASOs 440282M or 440223 but less than half as much increase in pre-mRNA levels was observed with ASO 440282 as observed in cells not treated with siRNA. Similar results were observed with ASO 440283 (data not shown).

## Discussion

In this paper we provide the first experimental evidence that certain ASOs designed to mediate RNase H1 cleavage of a targeted mRNA can, in certain instances, promote target reduction via non-RNase H mechanisms. We developed normal and splice-defective inducible minigenes and stably expressed them in order to evaluate the effects of mRNA processing on ASO activity (Figures 1, S1). The activities of 38 RNase H1-dependent ASOs were remarkably similar in normal and splice-defective transcripts (Figure 2A) with consistent differences in activity observed only with those ASOs targeted to intronic sequences. Previous studies have also suggested that introns that are less efficiently removed by the splicing machinery are more susceptible to ASO targeting than those that are spliced efficiently [31]; however, to our knowledge, this is the first time that this hypothesis has been tested directly. Interestingly, no intron ASOs were found to be active against the endogenous *SOD1* mRNA, suggesting that the endogenous pre-mRNA is processed more efficiently than the minigene transcript. This could be due to the fact that the intron in the minigene is truncated relative to the endogenous intron 4. Regions in both exons also showed slight differences in activity between the minigene and endogenous SOD1.

Levels of pre-mRNA were also assessed for both normal and splice-defective minigene transcripts (Figure 2B/C). Treatment of cells with ASOs targeting the region of the minigene transcript just downstream of the splice acceptor site significantly increased pre-mRNA levels in the control, but not the splice-defective construct. ASO targeting this region also most potently reduced levels of the spliced mRNA, and these ASOs had greater activity against the normal than the splice-defective construct. These data suggest that a portion of the spliced mRNA reduction in this region is independent of RNase H cleavage and related to the ability of the ASO to affect pre-mRNA processing and subsequent levels of the spliced message. Utilizing siRNAs to reduce RNase H1, we confirmed that a significant proportion of the activity of these ASOs was indeed RNase H1 independent. Whereas ASOs targeting other regions of the minigene transcript lost a significant amount of activity upon RNase H reduction, the effect of RNase H reduction on activity of ASOs downstream of the acceptor site was very modest (Figure 3A/C). We further demonstrated that ASO activity was independent of RNase H-mediated cleavage by treating cells with 2′MOE versions of the same ASOs. Fully 2′MOE modified ASOs have a very high affinity for targeted mRNA, are resist to both exo- and endonucleases, and do not support cleavage of hybridized mRNA by RNase H [32], [33]. In general, treatment with 2′MOE ASOs did not result in reduced levels of either spliced or pre-mRNA (Figure 4A); however, the 2′MOE ASOs targeting those sites unaffected by RNase H1 reduction did reduce levels of spliced mRNA. For example, a typical RNase H1-activating ASO 440222 reduced levels of both spliced and pre-mRNA in both control and splice-defective cell lines. In contrast, the 2′MOE analog, 440222M, had no effect on transcript levels (Figure 4B). In contrast, both RNase H1 and 2′MOE versions of 440282/3 increased pre-mRNA levels with concomitant reduction in spliced mRNA in the control cells line. In the splice-defective cell line some reduction of both spliced and pre-mRNA was observed; however, the level of reduction was less for cells treated with the 2′MOE ASO than with the gapmer ASO. These results indicate that the gapmer ASO reduces mRNA levels through both RNase H-dependent and independent mechanisms, whereas the 2′MOE works only by disrupting pre-mRNA processing. Consistent with this hypothesis, overexpression of *E. coli* RNase H in these cell lines had no effect on the activity of 2′MOE ASOs, but treatment with RNase H1-dependent ASOs no longer resulted in an increase pre-mRNA levels (Figure 4C). This is presumably because levels of endogenous RNase H1 are very low and rate limiting [5]. Highly abundant proteins that specifically bind an RNA/DNA heteroduplex might therefore be expected to limit or reduce access of RNase H1 and subsequent cleavage. Increasing the levels of RNase H by overexpression effectively shifted the competition toward cleavage. Overall reduction of spliced mRNA remained unchanged despite the lack of effect on the pre-mRNA likely due to the increase in RNase H cleavage activity.

Together, these data suggest a mechanism for ASOs 440282 and 440283 that reduces splicing efficiency by displacing hnRNPs, resulting in accumulation of pre-mRNA and ultimate reduction in spliced RNA levels. Mutation of these sites resulted in accumulation of the SOD/TO pre-mRNA (Figures 6 & S3), while reduction of hnRNP F, and to a lesser extent hnRNP H, reduced the rate of mRNA splicing of the minigene (Figure 7). It is interesting to note that mutation of the putative hnRNP binding sites also had a greater effect on hnRNP F binding than hnRNP H binding (Figure 6B). It has previously been shown that hnRNP proteins are associated with pre-mRNAs in the nucleus and regulate alternative splicing, polyadenylation, and other aspects of mRNA metabolism and transport [34]–[36]. We also showed that reduction of HSPA8 leads to an increase in potency of gapmer ASOs 440282 and 440283 (Figure 8). This suggests that HSPA8 may bind the ASO/RNA heteroduplex, preventing access to RNase H and subsequent cleavage (Figure 6B).

**Figure 9:**
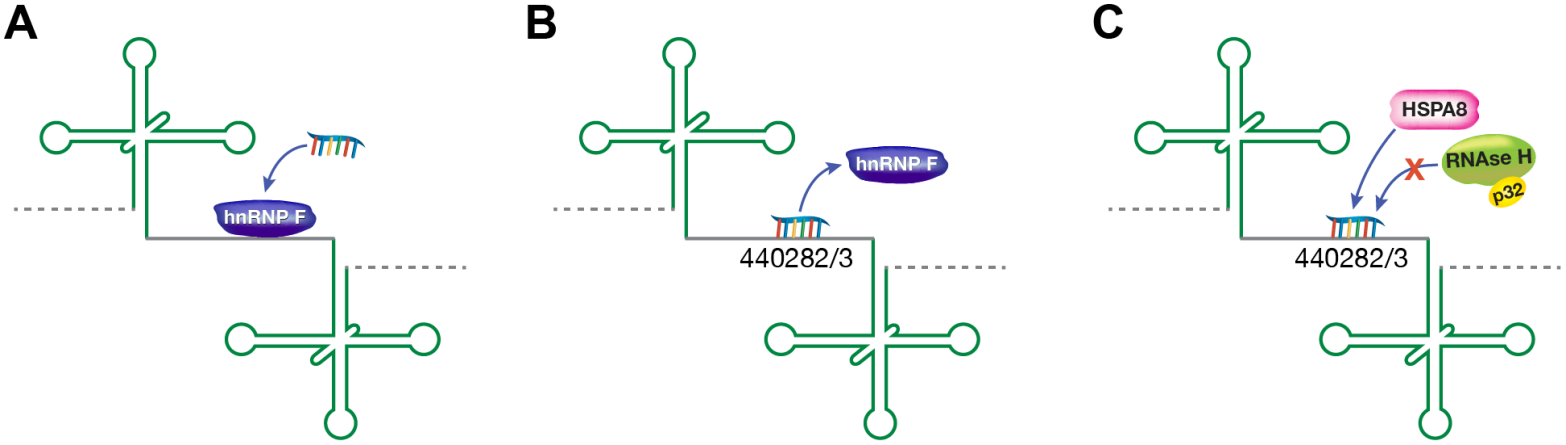
Proposed mechanism for non-RNase H activity with a DNA-like ASO. A) In the absence of ASO treatment hnRNPs bind the pre-mRNA, and it is efficiently spliced. B) A fully modified 2′MOE ASO binds the hnRNP site preventing hnRNP interaction with the pre-mRNA, leading to accumulation of pre-mRNA in the nucleus and reduction of spliced mRNA is the cytoplasm. C) An RNase H-activating ASO also competes with hnRNP for the binding site on the pre-mRNA; however, RNase H cleavage is prevented when HSPA8 binds the RNA/ASO heteroduplex.

The proposed mechanism for non-RNase H1 activity with a DNA-like ASO is illustrated in Figure 9. In the absence of ASOs, hnRNPs are recruited to the pre-mRNA, which is efficiently spliced (Figure 9A). Fully 2′MOE-modified ASOs bind pre-mRNA and prevent the recruitment of hnRNPs. In the absence of hnRNP binding, pre-mRNA accumulates and, as a result, overall levels of spliced mRNA decrease (Figure 9B). When RNase H1-activating ASOs bind the pre-mRNA, both RNase H-dependent and RNase H-independent mechanisms can decrease levels of transcript. In an RNase H-dependent route, the hnRNPs are displaced, RNase H1 is recruited, and the heteroduplex is cleaved (the ASO can also bind the spliced mRNA in the cytoplasm and recruit RNase H to cleave the heteroduplex), or the ASO can prevent hnRNP binding, operating through the same mechanism as 2′MOE ASOs. It is also possible that HSPA8 binds to the heteroduplex limiting access by splicing factors (Figure 9C). As with the 2′MOE ASO, in the absence of hnRNP binding, pre-mRNA accumulates and spliced mRNA decreases.

Given the rate of RNase H1 cleavage [37] and the affinity for the RNA/DNA heteroduplex [38] it was surprising that DNA-like ASOs reduce mRNA by a mechanism other than cleavage. siRNAs that promote cleavage of a targeted RNA through the RNA interference mechanism do so through an antisense mechanism very similar to that of RNase H-dependent ASOs. In contrast to the RNAi mechanism, where the siRNA oligonucleotide is bound to the cleavage enzyme prior to interacting with the target RNA [39], [40], RNase H1-dependent ASOs bind to the target RNA prior to interaction with the enzyme. It is likely that this difference allows for the competing mechanism that appears to be the result of steric inhibition of binding of splicing factors at a site required for efficient processing of the pre-mRNA, with RNase H-mediated cleavage prevented by binding of a second protein to the ASO/pre-mRNA heteroduplex.

ASOs designed to interact with RNA may target sequences that also play important roles in the intermediary metabolism of RNA and therefore may produce effects which altar RNA processing in addition to cleavage. For those ASO sequences that can activate multiple mechanisms, there does appear to be increased potency relative to gapmer ASOs that can activate RNAse H1 cleavage only. Clearly, further investigation will be required to determine whether competition between RNase H-mediated cleavage and occupancy mechanisms is universal. The results reported here suggest that targeting ASOs to sites such as those identified here that mediate mRNA reduction through mechanisms dependent on and independent of RNase H may result in increased potency relative to ASO that operate through a single mechanism. ASO targeted to this type of site on a transcript may ultimately reduce cost of treatment with ASO-based therapeutics.

## Supporting Information

Figure S1 A) Kinetics of SOD1 minigene pre-mRNA induction. SOD/TO cells were incubated in the presence of 1 µg/ml TET from 0 to 5 hours. Expression of pre-mRNA was evaluated using primer probe set E4 PRE. Expression is shown relative to basal pre-mRNA levels in the absence of TET induction. B) Kinetics of SOD1 minigene spliced mRNA induction was evaluated using primer/probe set E4 SPL. C) Minigene expression in SOD/TO constructs. TET was added to SOD/TO or SOD/TO-187 cells seeded in 6-well dishes at 1 µg/ml in DMEM, 10% FCS. Cells were harvested at 30, 60, and 160 minutes and RNA purified as detailed in Materials and Methods. Expression of minigene pre-mRNA and spliced mRNA was assessed by qRT/PCR using splice-specific primer/probe sets as detailed in Figure 1A. For the control minigene, addition of TET resulted in an almost 2 fold induction of spliced mRNA (Spliced +T) expression by 30 minutes, which increased to approximately 4 fold by 60 minutes and 8 fold by 160 minutes. In contrast levels of pre-mRNA induction (Pre +T) remained steady over all time points. D) TET addition to SOD/TO-187 minigene cells resulted in only low level induction of spliced mRNA that increased slightly over time; pre-mRNA levels were induced ∼2 fold at 30 minutes, ∼4 fold at 60 minutes, and ∼6 fold by 160 minutes. E) Spliced minigene mRNA is localized predominantly in the cytoplasm and pre-mRNA is observed in the nucleus of cells. Nuclear and cytoplasmic RNA was isolated from 5×106 cells treated for 60 minutes with TET using the Ambion PARIS kit according to the manufacturer’s protocol (Life Technologies). Expression of minigene pre-mRNA and spliced mRNA was assessed by qRT/PCR using splice-specific (E4 SPL) or pre-mRNA-specific (E4 PRE) primer probe sets. The bulk of SOD/TO minigene transcript was expressed as spliced mRNA (filled bars), the majority of which was present in the cytoplasm (grey bar). For the mutant mini-gene, SOD/TO-187, the bulk of the TET induced RNA transcript present was pre-mRNA (hatched bars), the majority of which was present in the nucleus (black hatched). F) Purity of nuclear (solid) and cytoplasmic (striped) fractions was evaluated by qRT/PCR using a primer/probe set for MALAT1 (nuclear) and PTEN (cytoplasmic).
(PDF)

Figure S2 A) RNA pull-down experiments performed essentially as detailed in Figure 5 except that both gapmer and full 2′MOE ASOs were used. B) Binding of proteins identified by RNA pull-down (Figure 5B) was confirmed by western blotting. Proteins released from the ASO/RNA complex were separated on 4–12% bis-Tris gels in 1X MOPS buffer then transferred to PVDF (Invitrogen). The membranes were blocked for 1 h in PBS containing 0.05% Tween 20 (PBST) and 5% milk powder. After overnight incubation at 4 C with indicated antibody, the membranes were washed in PBST and incubated with a 1/5000 dilution of goat anti-rabbit or goat anti-mouse HRP-conjugated antibody in blocking buffer. Membranes were washed and developed using ECL detection system (Amersham Biosciences). Antibodies were purchased from ABCAM: hnRNP K #ab52600; HSPA8 #ab79857; YBX1 #ab12148; GTF2I #ab88864; DHX36 ab70269; RNP-UL1 ab68480; hnRNP H #ab10374.
(PDF)

Figure S3 **TET-induced pre-mRNA levels for control and mutant SOD minigene.** SOD/TO or SOD/TO-AB cells were seeded in 96-well plates at ∼50% confluency. The following day minigene transcription was induced by addition of growth media containing 1 µg/ml TET. Cells were harvested at the indicated intervals and total RNA was purified using an RNeasy 3000 BioRobot. Pre-mRNA levels were assessed by qRT/PCR using primer/probe set E4 SPL as detailed in Figure 1. To avoid artifacts based upon well-to-well variation in cell number, mRNA levels were normalized to the total amount of RNA present in each reaction as determined by Ribogreen assay.
(PDF)

Figure S4 **RNAse H independent ASO activity in HeLa cells.** A) Full MOE ASOs are active at the 440283 site. HeLa cells with stably incorporated SOD/TO minigene were seeded in 96 well plates at 5000 cells/well. Cells were treated the following day with the indicated concentrations of full MOE ASOs 440283 (red lines) or 440247 (black lines) in Opti-MEM media (Invitrogen) containing 5 µg/ml Lipofectamine 2000 (Invitrogen) for 4 hours. Following transfection cells were washed 1X with PBS, then fed with fresh growth media containing 1 ug/ml tetracycline to induced minigene transcription. After 4 hours cells were harvested and expression of minigene spliced and pre-mRNA assessed by qRT/PCR. Data is plotted as percent mock-treated control for spliced mRNA (solid lines) and pre-mRNA (dashed lines). B) Gapmer ASO also increases pre-mRNA levels in HeLa cells. HeLa SOD/TO cells were treated as above with gapmer or full MOE ASOs at 30 nM. Expression of minigene spliced and pre-mRNA was assessed by qRT/PCR using primer/probes E4 SPL and E4 PRE. Data is plotted as percent expression relative to mock-treated control for spliced mRNA (solid bars) and pre-mRNA (lined bars).
(PDF)

Table S1 **Sequences of primers/probes used for qRT/PCR.** For primers complementary to the minigene, vector sequence is in lower case.
(PDF)
