# Supplementary figures and images for: Antisense Oligonucleotides Capable of Promoting Specific Target mRNA Reduction via Competing RNase H1-Dependent and Independent Mechanisms
Source: PLoS One. 2014 Oct 9;9(10):e108625. doi: 10.1371/journal.pone.0108625 (PMC4191969; doi:10.1371/journal.pone.0108625)

Figure S1

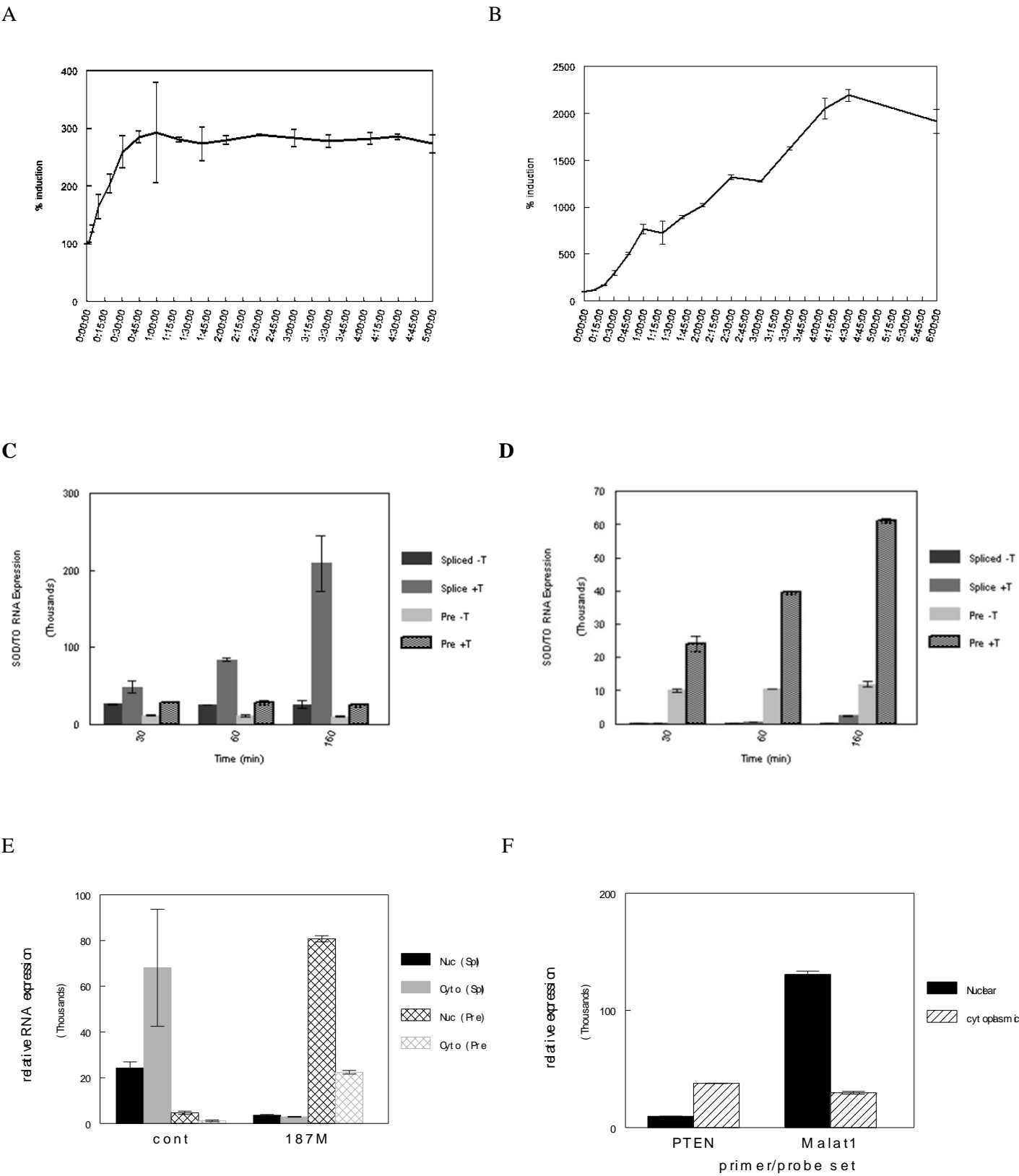

Supplement: Figure S1 — A) Kinetics of SOD1 minigene pre-mRNA induction. SOD/TO cells were incubated in the presence of 1 µg/ml TET from 0 to 5 hours. Expression of pre-mRNA was evaluated using primer probe set E4 PRE. Expression is shown relative to basal pre-mRNA levels in the absence of TET induction. B) Kinetics of SOD1 minigene spliced mRNA induction was evaluated using primer/probe set E4 SPL. C) Minigene expression in SOD/TO constructs. TET was added to SOD/TO or SOD/TO-187 cells seeded in 6-well dishes at 1 µg/ml in DMEM, 10% FCS. Cells were harvested at 30, 60, and 160 minutes and RNA purified as detailed in Materials and Methods. Expression of minigene pre-mRNA and spliced mRNA was assessed by qRT/PCR using splice-specific primer/probe sets as detailed in Figure 1A. For the control minigene, addition of TET resulted in an almost 2 fold induction of spliced mRNA (Spliced +T) expression by 30 minutes, which increased to approximately 4 fold by 60 minutes and 8 fold by 160 minutes. In contrast levels of pre-mRNA induction (Pre +T) remained steady over all time points. D) TET addition to SOD/TO-187 minigene cells resulted in only low level induction of spliced mRNA that increased slightly over time; pre-mRNA levels were induced ∼2 fold at 30 minutes, ∼4 fold at 60 minutes, and ∼6 fold by 160 minutes. E) Spliced minigene mRNA is localized predominantly in the cytoplasm and pre-mRNA is observed in the nucleus of cells. Nuclear and cytoplasmic RNA was isolated from 5×10^6^ cells treated for 60 minutes with TET using the Ambion PARIS kit according to the manufacturer’s protocol (Life Technologies). Expression of minigene pre-mRNA and spliced mRNA was assessed by qRT/PCR using splice-specific (E4 SPL) or pre-mRNA-specific (E4 PRE) primer probe sets. The bulk of SOD/TO minigene transcript was expressed as spliced mRNA (filled bars), the majority of which was present in the cytoplasm (grey bar). For the mutant mini-gene, SOD/TO-187, the bulk of the TET induced RNA transcript present was pre-m [file pone.0108625.s001.pdf]

Figure S2

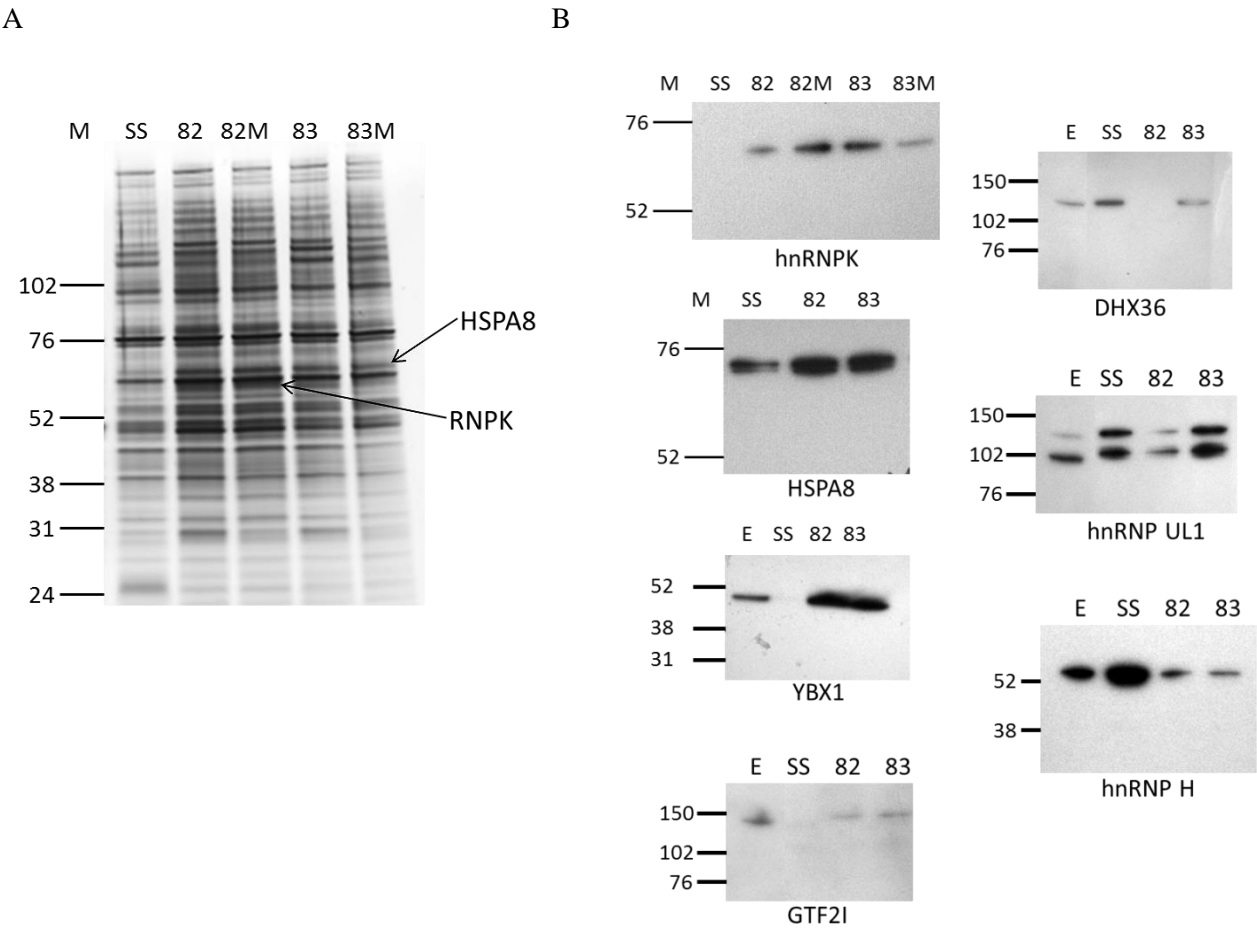

Supplement: Figure S2 — A) RNA pull-down experiments performed essentially as detailed in Figure 5 except that both gapmer and full 2′MOE ASOs were used. B) Binding of proteins identified by RNA pull-down (Figure 5B) was confirmed by western blotting. Proteins released from the ASO/RNA complex were separated on 4–12% bis-Tris gels in 1X MOPS buffer then transferred to PVDF (Invitrogen). The membranes were blocked for 1 h in PBS containing 0.05% Tween 20 (PBST) and 5% milk powder. After overnight incubation at 4 C with indicated antibody, the membranes were washed in PBST and incubated with a 1/5000 dilution of goat anti-rabbit or goat anti-mouse HRP-conjugated antibody in blocking buffer. Membranes were washed and developed using ECL detection system (Amersham Biosciences). Antibodies were purchased from ABCAM: hnRNP K #ab52600; HSPA8 #ab79857; YBX1 #ab12148; GTF2I #ab88864; DHX36 ab70269; RNP-UL1 ab68480; hnRNP H #ab10374. (PDF) [file pone.0108625.s002.pdf]

Figure S3

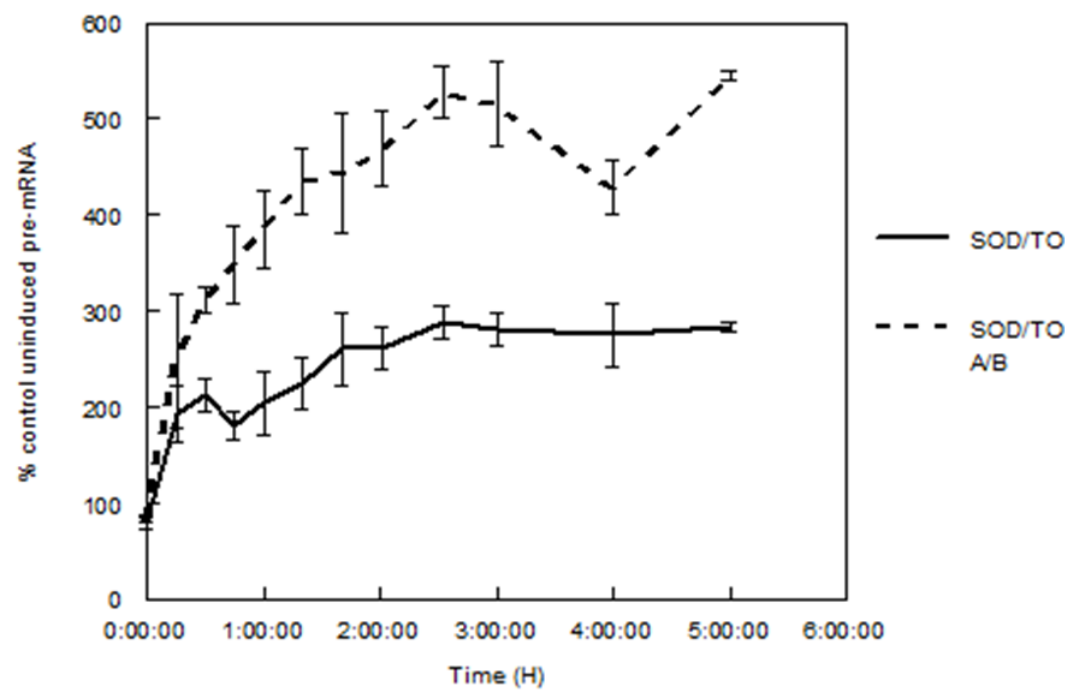

Supplement: Figure S3 — TET-induced pre-mRNA levels for control and mutant SOD minigene. SOD/TO or SOD/TO-AB cells were seeded in 96-well plates at ∼50% confluency. The following day minigene transcription was induced by addition of growth media containing 1 µg/ml TET. Cells were harvested at the indicated intervals and total RNA was purified using an RNeasy 3000 BioRobot. Pre-mRNA levels were assessed by qRT/PCR using primer/probe set E4 SPL as detailed in Figure 1. To avoid artifacts based upon well-to-well variation in cell number, mRNA levels were normalized to the total amount of RNA present in each reaction as determined by Ribogreen assay. (PDF) [file pone.0108625.s003.pdf]

Figure S4

A

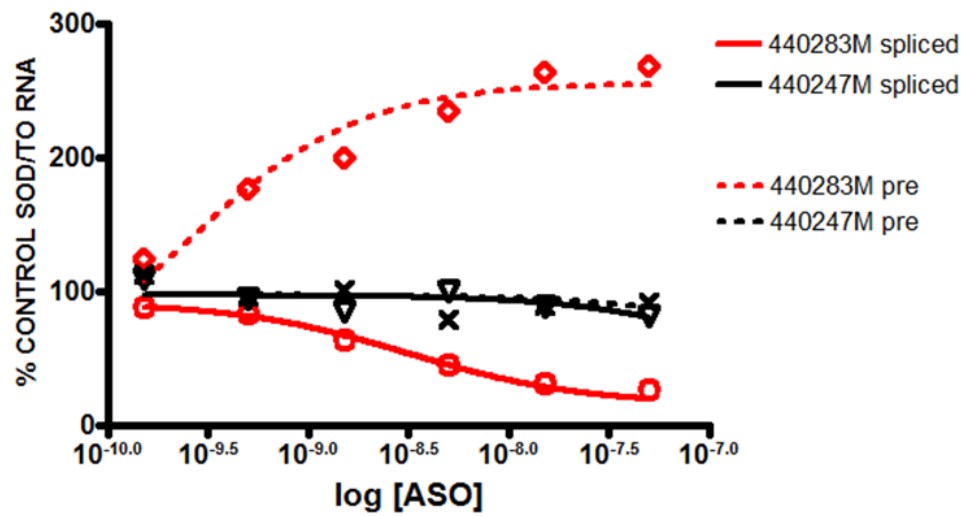

B

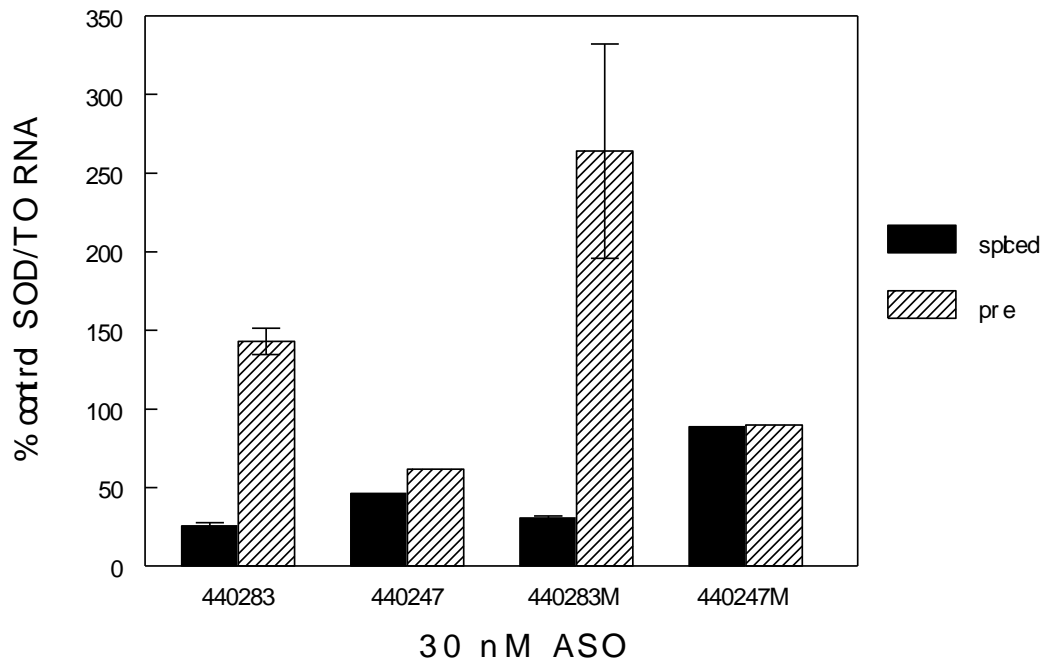

Supplement: Figure S4 — RNAse H independent ASO activity in HeLa cells. A) Full MOE ASOs are active at the 440283 site. HeLa cells with stably incorporated SOD/TO minigene were seeded in 96 well plates at 5000 cells/well. Cells were treated the following day with the indicated concentrations of full MOE ASOs 440283 (red lines) or 440247 (black lines) in Opti-MEM media (Invitrogen) containing 5 µg/ml Lipofectamine 2000 (Invitrogen) for 4 hours. Following transfection cells were washed 1X with PBS, then fed with fresh growth media containing 1 ug/ml tetracycline to induced minigene transcription. After 4 hours cells were harvested and expression of minigene spliced and pre-mRNA assessed by qRT/PCR. Data is plotted as percent mock-treated control for spliced mRNA (solid lines) and pre-mRNA (dashed lines). B) Gapmer ASO also increases pre-mRNA levels in HeLa cells. HeLa SOD/TO cells were treated as above with gapmer or full MOE ASOs at 30 nM. Expression of minigene spliced and pre-mRNA was assessed by qRT/PCR using primer/probes E4 SPL and E4 PRE. Data is plotted as percent expression relative to mock-treated control for spliced mRNA (solid bars) and pre-mRNA (lined bars). (PDF) [file pone.0108625.s004.pdf]
