# Supplementary material for: Antisense Oligonucleotides Capable of Promoting Specific Target mRNA Reduction via Competing RNase H1-Dependent and Independent Mechanisms
Source: PLoS One. 2014 Oct 9;9(10):e108625. doi: 10.1371/journal.pone.0108625 (PMC4191969; doi:10.1371/journal.pone.0108625)
Supplement: Table S1 — Sequences of primers/probes used for qRT/PCR. For primers complementary to the minigene, vector sequence is in lower case. (PDF) [file pone.0108625.s005.pdf]

Table S1

Target RNA	Forward primer	Reverse primer	probe
SOD mini E4 SPL	taa tac gac tca cta tag gga ga	CTG CTT TTT CAT GGA CCA CCA	CAA AGA TGG TGT GGC CGA TG
SOD mini E5 SPL	TGG TGG TCC ATG AAA AAG CAG	ctg tgc tgg ata tct gca gaa ttc TTT AG	CGC TGG AAG TCG TTT GGC TTG TGG
SOD mini E4 PRE	taa tac gac tca cta tag gga ga	GGA TCT TTA GAA ACC GCG ACT	CAA AGA TGG TGT GGC CGA TG
SOD mini E5 PRE	TGG TGG TCC ATG AAA AAG CAG	TGG GTA TTG TTG GGA GGA GGT A	CGC TGG AAG TCG TTT GGC TTG TGG
SOD1 (endo)	TGCAGGGCATCATC AATTTC	AGTCAGTCCTTTAATG CTTCCCC	AGCAGAAGGAAAGTAA TGGACCAGTGAAGGTG
RNAse H1	CCTGTACTTACTGG TGTGGAAAATAGC	CCGTGTGAAAGACGC ATCTG	TGCAGGTAGGACCATT GCAGTGATGG
PTEN	AATGGCTAAGTGA AGATGACAATCAT	TGCACATATCATTACA CCAGTTCGT	TTGCAGCAATTCACTGT AAAGCTGGAAAGG
MALAT1	AGGCGTTGTGCGTA GAGGAT	AAAGGTTACCATAAGT AAGTTCCAGAAAA	AGTGGTTGGTAAAAAT CCGTGAGGTCGG
